# Opioid-sparing anesthesia with dexmedetomidine provides stable hemodynamic and short hospital stay in non-intubated video-assisted thoracoscopic surgery: a propensity score matching cohort study

**DOI:** 10.1186/s12871-023-02032-0

**Published:** 2023-04-03

**Authors:** Hui-Hsuan Ke, Jing-Yang Liou, Wei-Nung Teng, Po-Kuei Hsu, Mei-Yung Tsou, Wen-Kuei Chang, Chien-Kun Ting

**Affiliations:** 1 Department of Anesthesiology, School of Medicine, National Yang Ming Chiao Tung University, Taipei, Taiwan; 2 Department of Anesthesiology, Taipei Veterans General Hospital, Taipei, Taiwan; 3 Department of Surgery, Division of Thoracic Surgery, Taipei Veterans General Hospital, Taipei, Taiwan

**Keywords:** Dexmedetomidine, Non-intubated video-assisted thoracic surgery, Opioid-related complications, Hemodynamic stability, Hospital stay

## Abstract

**Objectives:**

Dexmedetomidine is an alpha-2 agonist with anti-anxiety, sedative, and analgesic effects and causes a lesser degree of respiratory depression. We hypothesized that the use of dexmedetomidine in non-intubated video-assisted thoracic surgery (VATS) may reduce opioid-related complications such as postoperative nausea and vomiting (PONV), dyspnea, constipation, dizziness, skin itching, and cause minimal respiratory depression, and stable hemodynamic status.

**Methods:**

Patients who underwent non-intubated VATS lung wedge resection with propofol combined with dexmedetomidine (group D) or alfentanil (group O) between December 2016 and May 2022 were enrolled in this retrospective propensity score matching cohort study. Intraoperative vital signs, arterial blood gas data, perioperative results and treatment outcomes were analyzed.

Of 100 patients included in the study (group D, 50 and group O, 50 patients), group D had a significantly lower degree of decrement in the heart rate and the blood pressure than group O. Intraoperative one-lung arterial blood gas revealed lower pH and significant ETCO_2_. The common opioid-related side effects, including PONV, dyspnea, constipation, dizziness, and skin itching, all of which occurred more frequently in group O than in group D. Patients in group O had significantly longer postoperative hospital stay and total hospital stay than group D, which might be due to opioid-related side effects postoperatively.

**Conclusions:**

The application of dexmedetomidine in non-intubated VATS resulted in a significant reduction in perioperative opioid-related complications and maintenance with acceptable hemodynamic performance. These clinical outcomes found in our retrospective study may enhance patient satisfaction and shorten the hospital stay.

## Introduction

Anesthesia administered during non-intubated video-assisted thoracic surgery (VATS) includes both sedation and regional analgesia [1, 2]. However, excessive opioid administration during and after surgery has been known to cause nausea, vomiting, constipation, tolerance, and respiratory depression [3]. Abnormal breathing during non-intubated VATS can lead to low blood pH, CO_2_ accumulation, and paradoxical respiration, thereby jeopardizing the safety of the surgical field.

Dexmedetomidine is an alpha-2 agonist with anti-anxiety, sedative, and analgesic effects and causes a lesser degree of respiratory depression than propofol [4]. During surgery, dexmedetomidine provides effective perioperative analgesia and reduces opioid requirements without increasing the incidence of side effects, especially respiratory depression [5]. Dexmedetomidine may also preserve the protective mechanism of hypoxic pulmonary vasoconstriction (HPV) and improve arterial oxygenation during one lung ventilation in adult patients undergoing thoracic surgery [6].

We hypothesized that the use of dexmedetomidine may reduce opioid-related complications such as postoperative nausea and vomiting (PONV), dyspnea, constipation, dizziness, skin itching, and cause minimal respiratory depression, and stable hemodynamic status. The aims of reducing opioid-related complications by using dexmedetomidine are to provide high-quality and safe intraoperative care, improve patient’s postoperative satisfaction, as well as shorten postoperative hospital stay by reducing opioid-related complications.

## Materials and methods

The study protocol was reviewed and approved by the Institutional Review Board (IRB) of Taipei Veterans General Hospital in Taiwan (approval number: 2017–01-016 AC). Since this was a retrospective propensity score matching cohort study, the need for informed consent was waived. We checked medical records one by one and excluded patients with incomplete data. Patients who underwent non-intubated VATS lung wedge resection with propofol combined with dexmedetomidine (*n* = 50) or alfentanil (*n* = 50) between December 2016 and May 2022 were enrolled. The surgeries were performed by a single thoracic surgical team with uniportal thoracoscopic wedge resection surgery.

### Anesthesia procedures

The patients were preoxygenated using transnasal humidified rapid-insufflation ventilatory exchange (THRIVE) prior to anesthesia at an initial flow rate of 30 L/min. Pulse oximetry, electrocardiography, blood pressure with invasive radial artery monitoring and frontal Bispectral Index™ (BIS™) monitoring were performed during surgery. A CO_2_ sampling line was inserted into one of the nostrils to monitor end-tidal carbon dioxide (ETCO_2_).

One group received IV propofol by target-controlled infusion and dexmedetomidine pump (Group D), while the other group received IV propofol by target-controlled infusion and bolus alfentanil (Group O). Patients were sedated with either one of the two anesthesia regimens, selected by the anesthesiologist on duty. The detailed protocols of both groups were as follows:

In group D, patients were premedicated with 2 mg of intravenous (IV) midazolam and loading dose of dexmedetomidine (0.5 μg/kg for 30 min). Next, thoracic epidural catheter was inserted between T7 and T8 for regional anesthesia, and a test dose of 2 ml of 2% lidocaine was administered for confirmation. Lidocaine 150 mg and fentanyl 50 μg were loaded via epidural catheter for analgesia, and bupivacaine 0.25% was administered for maintenance according to the patient’s blood pressure. After preoxygenation and premedication, pre-one lung arterial blood gas data were obtained. The patients were then maintained with IV propofol by target-controlled infusion and maintenance dose of dexmedetomidine (0.5 μg/kg/hr) until the surgery was finished.

In group O, patients were premedicated with 2 mg of intravenous (IV) midazolam, a thoracic epidural catheter was inserted between T7 and T8 for regional anesthesia, and a test dose of 2 ml of 2% lidocaine was administered for confirmation. Lidocaine 150 mg and fentanyl 50 μg were loaded via epidural catheter for analgesia, and bupivacaine 0.25% was administered for maintenance according to the patient’s blood pressure. After preoxygenation and premedication, pre-one lung arterial blood gas data were obtained, followed by maintenance with IV propofol by target-controlled infusion and bolus alfentanil. The purpose of bolus alfentanil was to offer pain control, lower the consumption of propofol for sedation and to control the patient’s respiratory rate, because rapid and shallow breathing might interfere the operation field.

During VATS, the patients were placed in the lateral decubitus position. The THRIVE was adjusted to a flow rate of 30–50 L/min (FiO_2_ = 1.0) to maintain oxygen saturation and wash out CO_2_ to prevent excessive hypercapnia.

Routine intraoperative monitoring was performed, and the BIS value was maintained between 40 and 60. The incision was covered with a wound protector/retractor. An iatrogenic pneumothorax was created, and the lung was collapsed while spontaneous respiration was maintained with one-lung ventilation.

An internal intercostal nerve block was performed under thoracoscopic guidance by injecting 1 to 2 mL bupivacaine 0.25% from the second to the eighth intercostal spaces, and then lung wedge resection was completed by the chief surgeon. Finally, a pigtail catheter was inserted through the thoracoscopic incision wound and the Thopaz Digital Chest Drainage System (THOPAZ) was connected. The pigtail catheter was removed in the postoperative room after checking for no signs of air leakage on postoperative chest radiography. The epidural catheter was removed after 50 μg of fentanyl was administered for postoperative analgesia.

### PONV and common opioid-related side effects

PONV, defined as occurring during the first 24 to 48 hours after surgery, is the most frequent side effect after general anesthesia and the least desirable outcome following surgery. We evaluated PONV moderate to high-risk patients with Apfel score before the surgery and gave prokinetic premedication, such as dexamethasone 4 mg for patients who had 1–2 risk factors, and dexamethasone 4 mg plus metoclopramide 5 mg for patients with 3 risk factors (There was no patients with 4 risk factors in this study). PONV and other common opioid-related side effects, including dyspnea, constipation, dizziness and skin itching were recorded in our study.

### Intra- and postoperative pain management

Our protocol was designed to control pain by multiple medication. First, dexmedetomidine has been proven to reduce perioperative opioid consumption and postoperative pain intensity. On the other hand, we applied intraoperative loading, postoperative single-dose fentanyl (50 μg), and bupivacaine 0.25% for maintenance through thoracic epidural analgesia, intraoperative intercostal nerve block as well as postoperative opioid and adjuvant analgesics, such as tramal, Ultracet® tablets, Traceton® tablets, and NSAIDs (parecoxib, diclofenac tablets). Morphine was administered in the postoperative room in our regimen when the numerical rating scale was rated higher than three points by patients.

### Data collection and statistical analysis

Demographic data and surgical outcomes were retrospectively obtained by reviewing medical charts. Anesthesia data, including oxygen saturation, preoperative and intraoperative arterial blood gas data (partial pressure of carbon dioxide [PaCO_2_], partial pressure of oxygen [PaO_2_], and peripheral oxygen saturation), heart rate, and blood pressure, were obtained from the anesthetic records.

Statistical analyses were performed using the IBM SPSS Statistics V26.0. Categorical data were analyzed using propensity score matching (PSM), paired sample t-test, cross-tabulation, and chi-square test. Numerical data are presented as mean ± standard deviation.

Propensity score matching (PSM) analysis was performed with the matching package in R software (version 4.0.2 for Windows, Bell Laboratories) and conducted with the 1:1 nearest neighbor matching method. The covariates included all patient characteristics, such as sex, age, BMI, ASA classification, pre-op lung condition, smoker, diagnosis, and comorbidities. Statistical significance was set at *p* value < 0.05.

## Results

In this retrospective study, the initial amount of group D was 58 patients during our data collecting period. We excluded 8 patients with incomplete medical records. After PSM analysis, patients with propofol combined with dexmedetomidine (*n* = 50) or alfentanil (*n* = 50) between December 2016 and May 2022 were enrolled. The characteristics of patients undergoing video-assisted thoracic surgery with dexmedetomidine and propofol (group D, *n* = 50) or alfentanil and propofol (group O, *n* = 50) are shown in Table 1.

**Table 1:** Characteristics of patients who underwent video-assisted thoracic surgery

Variables	Group D (*N* = 50)	Group O (*N* = 50)	*p*
Male	18 (36)	27 (54)	0.07
Female	32 (64)	23 (46)	
Age (years)	55.1 ± 12.1	57.1 ± 16	0.68
Height (cm)	162.1 ± 7.3	163.4 ± 8.1	0.8
Weight (kg)	61.3 ± 11.5	62.3 ± 10.4	0.34
BMI (kg/m^2^)	23.2 ± 3.1	23.3 ± 3.1	0.77
ASA classification			
I	7 (14)	4 (8)	0.37
II	38 (76)	37 (74)	
III	5 (10)	9 (18)	
Pre-op lung condition			
FEV_1_/FVC (% of prediction)	83.8 ± 7.1	83.7 ± 8.1	0.92
Smoker	3 (6)	4 (8)	0.7
Diagnosis			
Adenocarcinoma	29 (58)	21 (42)	0.43
Metastatic lung cancer	12 (24)	15 (30)	
Benign lung tumor	1 (2)	2 (4)	
Others	8 (16)	12 (24)	
Comorbidity			
No systemic disease	7 (14)	10 (20)	0.42
Hepatitis carrier	6 (12)	7 (14)	0.77
Extrapulmonary tumor	15 (30)	22 (44)	0.15
Cardiac disease	6 (12)	5 (10)	0.75
Hypertension	15 (30)	13 (26)	0.66
Diabetes mellitus	7 (14)	5 (10)	0.54
Pulmonary disease (COPD, asthma)	2 (4)	3 (6)	0.65

Continuous data are presented as the mean ± standard deviation, and categorical data are presented as n (%)

^a^*p* < 0.05 indicates a significant difference between groups D and O

Key: *ASA* American Society of Anesthesiologists, *BMI* Body mass index, *COPD* Chronic obstructive pulmonary disease, *DLCO* Diffusing capacity for carbon monoxide, *FEV1* Forced expiratory volume-one second, *FVC* Forced vital capacity

**Fig. 1:**
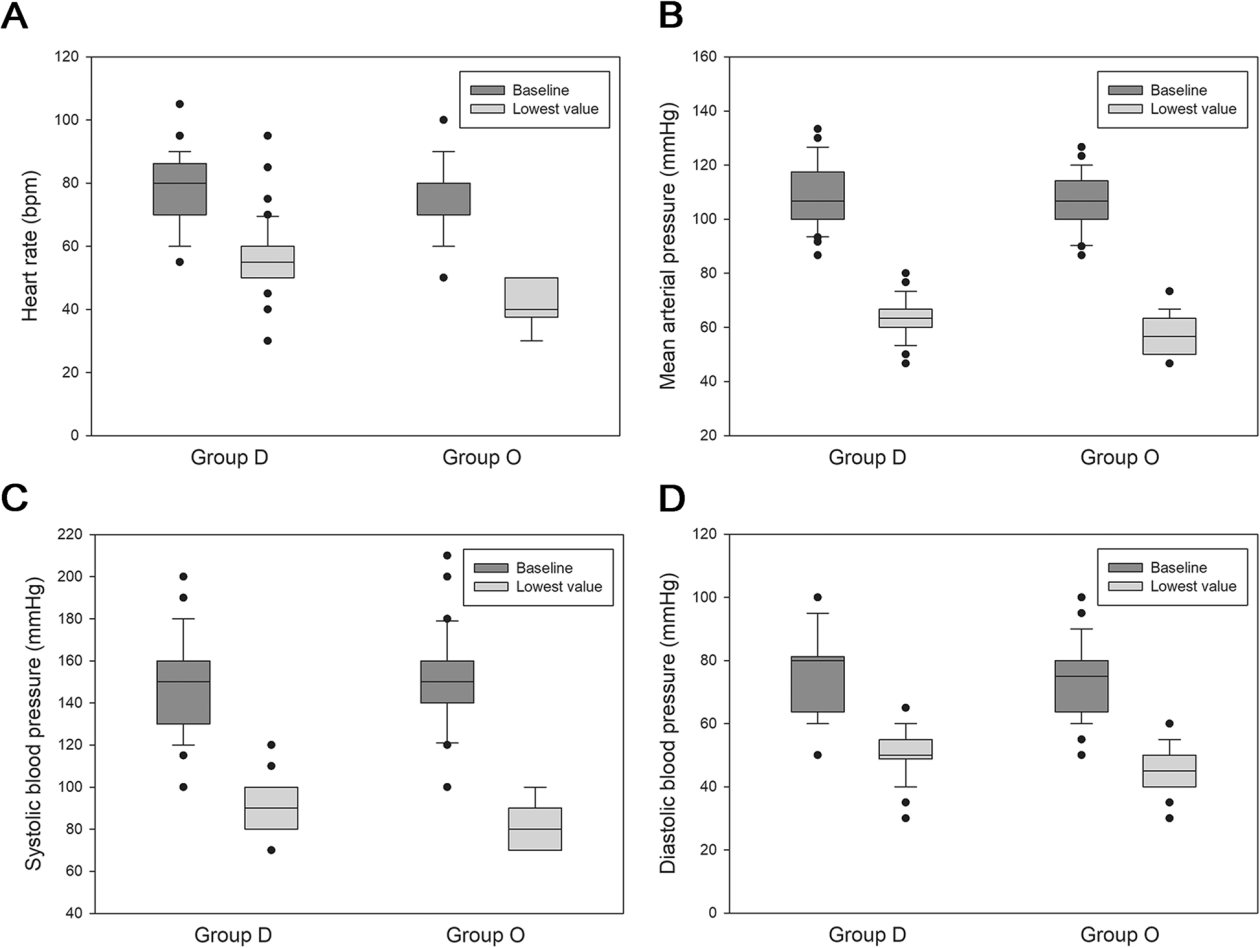
Intraoperative vital signs. **A** Intraoperative heart rate. **B** Intraoperative mean arterial pressure. **C** Intraoperative systolic blood pressure. **D** Intraoperative diastolic blood pressure

**Fig. 2:**
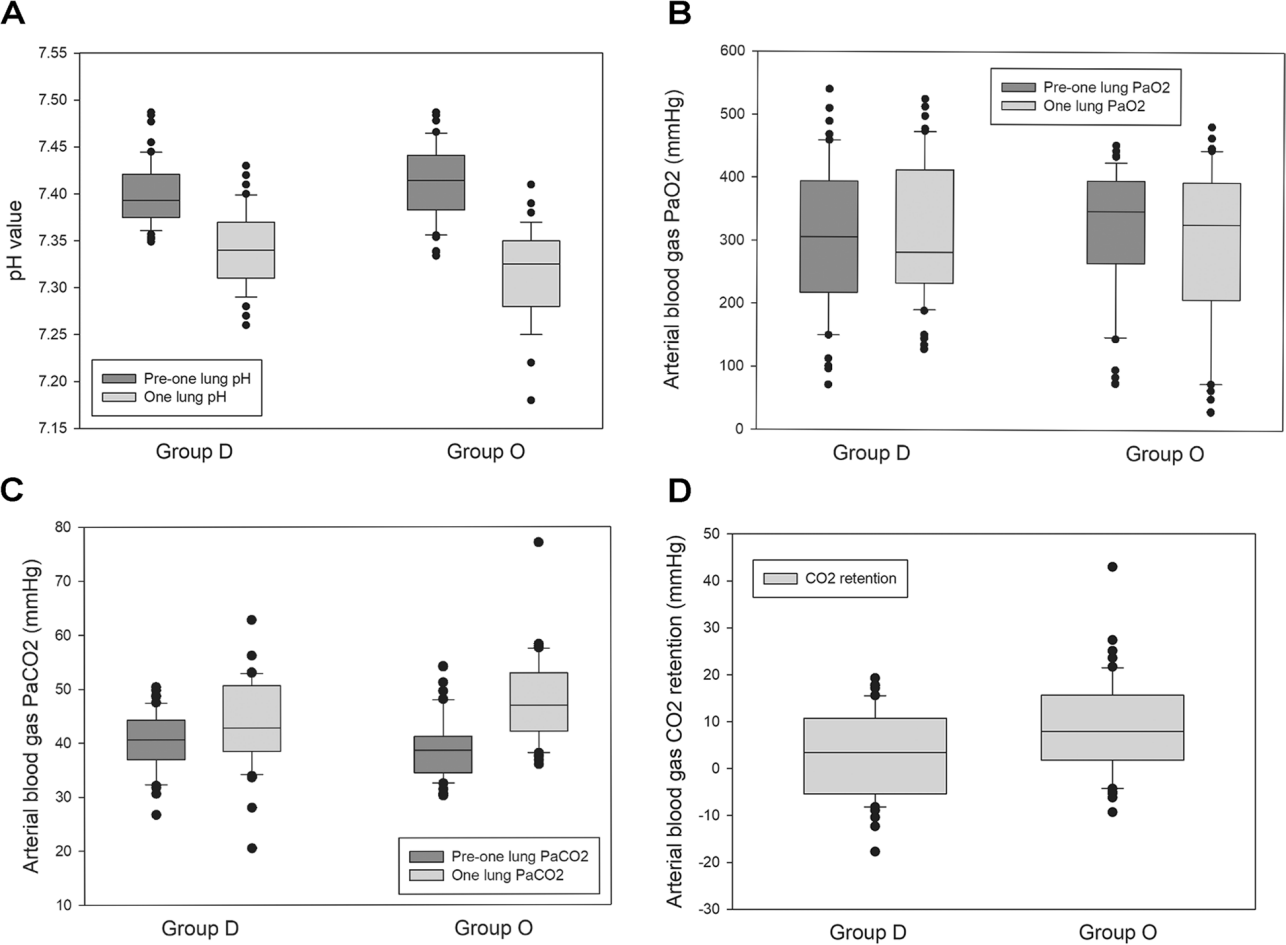
Intraoperative arterial blood gas. **A** Intraoperative pH. **B** Intraoperative PaO_2_. C. Intraoperative PaCO_2_. D. Intraoperative CO_2_ retention

**Fig. 3:**
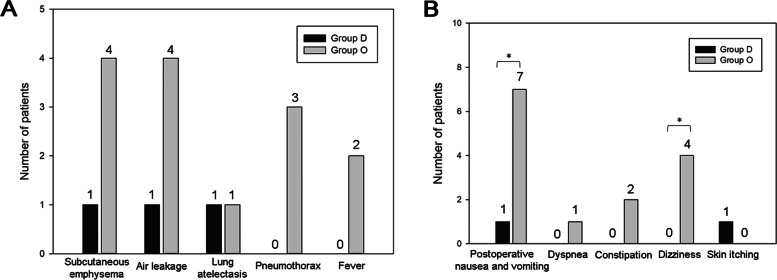
Perioperative results. **A** Postoperative complications. **B** Common opioid-related side effects. Asterisk (*) indicates a statistically significant difference (*p* < 0.05) between groups D and O

**Fig. 4:**
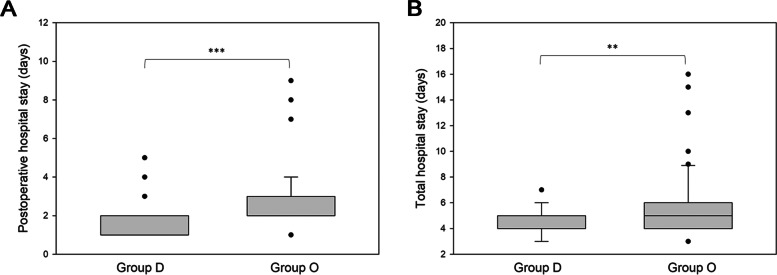
Hospital stay. **A** Postoperative hospital stay. **B** Total hospital stay. Asterisk (**) indicates a high statistically significant difference (*p* < 0.01) between groups D and O. Asterisk (***) indicates an extreme statistically significant difference (*p* < 0.001) between groups D and O

Table 2 and Figs. 1, 2, 3 and 4 represent the heart rate, blood pressure, and arterial blood gas data that were recorded and analyzed. Baseline heart rate was 77.7 ± 11 bpm in group D and 76.8 ± 11.1 bpm in group O. Intraoperative values were decreased in both groups, with group O showing a greater decrease than group D (group D vs groups O: 26.8 ± 12.2 vs 46.7 ± 9.8%, *p* < 0.05). Baseline systolic blood pressure, diastolic pressure, mean arterial blood pressure, and their lowest intraoperative values were recorded. Group D had a more maintained blood pressure than group O, with a significantly lower degree of decrement in the three blood pressure parameters.

**Table 2:** Intraoperative vital signs and arterial blood gas data

Variables	Group D (*N* = 50)	Group O (*N* = 50)	*p*
Heart rate			
Baseline value (bpm)	77.7 ± 11	76.8 ± 11.1	0.64
Lowest value (bpm)	56.4 ± 10.6	40.6 ± 7.4	< 0.001^c^
Decreased percentage from baseline level (%)	26.8 ± 12.2	46.7 ± 9.8	< 0.001^c^
Systolic blood pressure			
Baseline value (mmHg)	148 ± 22.9	150.2 ± 20.9	0.59
Lowest value (mmHg)	89.7 ± 11.5	83.6 ± 10.5	< 0.05^a^
Decreased percentage from baseline level (%)	38.1 ± 11.2	43.5 ± 9.7	< 0.01^b^
Diastolic blood pressure			
Baseline value (mmHg)	76.1 ± 13.7	73.8 ± 11.9	0.28
Lowest value (mmHg)	50 ± 7.6	45.4 ± 7.3	< 0.01^b^
Decreased percentage from baseline level (%)	33.1 ± 11.5	37.4 ± 11.4	< 0.05^a^
Mean blood pressure			
Baseline value (mmHg)	109.1 ± 11.6	105.8 ± 10.3	0.14
Lowest value (mmHg)	63.2 ± 7.7	58.1 ± 7.2	< 0.01^b^
Decreased percentage from baseline level (%)	42 ± 5.3	44.9 ± 5.2	< 0.01^b^
In room SpO_2_ (%)	98.1 ± 2	97.9 ± 2	0.56
Arterial blood gas data pre-one lung ventilation			
pH	7.4 ± 0	7.4 ± 0	0.07
PaO_2_ (mmHg)	305.3 ± 117.6	323.2 ± 102	0.41
PaCO_2_ (mmHg)	40.8 ± 5.8	38.9 ± 5.6	0.05
Saturation (%)	99.3 ± 1	99.4 ± 1.3	0.45
Arterial blood gas data during one-lung ventilation			
pH	7.34 ± 0	7.31 ± 0.1	< 0.01^b^
PaO_2_ (mmHg)	313 ± 110	291.3 ± 134	0.38
PaCO_2_ (mmHg)	44.1 ± 7.8	47.6 ± 7.8	< 0.05^a^
Saturation (%)	99.2 ± 0.9	99.1 ± 2.1	0.71
CO_2_ retention pre-one lung and one-lung ventilation (mmHg)	3.5 ± 9.2	8.8 ± 10.1	< 0.05^a^

Continuous data are presented as the mean ± standard deviation, and categorical data are presented as n (%)

^a^*p* < 0.05 indicates a significant difference between groups D and O

^b^*p* < 0.01, indicates a highly significant difference between groups D and O

^c^*p* < 0.001 indicates an extremely significant difference between groups D and O

Key: *PaO*_*2*_ Partial pressure of oxygen, *PaCO*_*2*_ Partial pressure of carbon dioxide, *SpO*_*2*_ Saturation of peripheral oxygen

There was no significant difference in preoperative in room SpO_2_ and pre-one lung ventilation arterial blood gas values of group D and group O, with SpO_2_: 98.1 ± 2 vs 97.9 ± 2, pH: 7.4 vs 7.4, PaO_2_: 305.3 ± 117.6 vs 323.2 ± 102, PaCO_2_: 40.8 ± 5.8 vs 38.9 ± 5.6 mmHg, and saturation: 99.3 ± 1 vs 99.4 ± 1.3%, respectively. On the other hand, intraoperative one-lung arterial blood gas in group D and group O revealed pH: 7.34 vs 7.31 ± 0.1 (*p* < 0.05), PaO_2_: 313 ± 110 vs 291.3 ± 134 mmHg, PaCO_2_: 44.1 ± 7.8 vs 47.6 ± 7.8 mmHg (*p* < 0.05), and saturation: 99.2 ± 0.9 vs 99.1 ± 2.1%. CO_2_ retention was 3.5 ± 9.2 mmHg in group D and 8.8 ± 10.1 mmHg in group O (*p* < 0.05).

Table 3 shows perioperative results and treatment outcomes. The average anesthetic induction duration was significantly longer in group D than group O (26.9 ± 9.4 vs 22.2 ± 9.2 min, *p* < 0.05), owing to the loading time of dexmedetomidine. Blood losses in all the cases were less than 30 ml and there was no conversion to thoracotomy.

**Table 3:** Perioperative results and treatment outcomes

Variables	Group D (*N* = 50)	Group O (*N* = 50)	*p*
Anesthetic induction duration (min)	26.9 ± 9.4	22.2 ± 9.2	< 0.05^a^
Bupivacaine consumption via epidural catheter (mg)	14.5 ± 3	13.7 ± 2.7	0.54
Propofol consumption (mg)	294.1 ± 34.1	324.5 ± 39.1	0.08
Intraoperative blood loss (ml)	< 30	< 30	1.00
Intraoperative complication	0 (0)	0 (0)	1.00
Conversion to thoracotomy	0 (0)	0 (0)	1.00
Intraoperative alfentanil consumption (μg)		742 ± 339.3	
Postoperative pain control			
Morphine consumption (mg)	1.1 ± 1.8	2.4 ± 3.8	< 0.05^a^
Parecoxib (Dynastat®) (mg)	31.2 ± 16.7	27.2 ± 29.6	0.42
Tramal (mg)	2 ± 14.1	2 ± 14.1	1
Ultracet® (tab)	1.08 ± 2.53	0.86 ± 0	0.55
Diclofenac (tab)	0 ± 0.1	0.5 ± 1.6	< 0.01^b^
Tramadol 37.5 mg + Acetaminophen 325 mg (Traceton®) (tab)	0	0.4 ± 1	< 0.01^b^
Postoperative hospital stay (days)	1.8 ± 0.8	2.9 ± 1.6	< 0.001^c^
Total hospital stay (days)	4.5 ± 0.9	5.8 ± 2.7	< 0.01^b^

Continuous data are presented as the mean ± standard deviation, and categorical data are presented as n (%)

^a^*p* < 0.05 indicates a significant difference between groups D and O

^b^*p* < 0.01 indicates a highly significant difference between groups D and O

^c^*p* < 0.001 indicates an extremely significant difference between groups D and O

Key: *PONV* Postoperative, nausea and vomiting

The average dose of intraoperative intravenous alfentanil was 742 ± 339.3 μg in group O. Regarding postoperative pain control, a significantly larger dose of morphine was needed in group O than group D (group D vs group O: 1.1 ± 1.8 vs 2.4 ± 3.8 mg, *p* < 0.05). Additionally, group O showed a larger consumption of nonsteroidal anti-inflammatory drugs (NSAIDs) diclofenac and Traceton®.

Fourteen patients in group O had postoperative complications, including subcutaneous emphysema, air leakage, lung atelectasis, pneumothorax, and fever, whereas three patients in group D had postoperative complications (Fig. 3A). We investigated the common opioid-related side effects, including PONV, dyspnea, constipation, dizziness, and skin itching, all of which occurred more frequently in group O than in group D (group D vs. group O: 2 vs. 14 patients) (Fig. 3B). Patients in group O had significantly longer postoperative hospital stay (group D vs group O 1.8 ± 0.8 vs 2.9 ± 1.6 days, *p* < 0.05) (Fig. 4A) and total hospital stay (group D vs group O 4.5 ± 0.9 vs 5.8 ± 2.7 days, *p* < 0.05) (Fig. 4B) than group D, which might be due to opioid-related side effects postoperatively.

## Discussion

The assessment of risk related to lung resection is a complex process that considers the results of patient pre-op tests, intervention type, age, and other risk factors. A multidisciplinary team that includes the specialists and anesthesiologists should be involved in managing the patient, assessing the procedure risk, optimizing the perioperative conditions, and planning the appropriate treatment regimens to minimize postoperative complications and length of hospital stay [7]. In our hospital, surgeons and anesthesiologist have reached a consensus, and our indication of conducting non-intubated VATS is consistent with the world trends [8, 9].

Dexmedetomidine is a highly selective α2-adrenergic receptor agonist associated with sedative and analgesic sparing effects, reduced delirium and agitation, perioperative sympatholysis, cardiovascular stabilizing effects, and preservation of respiratory function. Therefore, it has been successfully employed as a total anesthetic agent in day care minimally invasive procedures and other minor procedures facilitating early discharge [10, 11]. In 2006, Lee et al. reported that intraoperative dexmedetomidine administration could improve the quality of recovery, postoperative pulmonary function, and other outcomes, and reduce hospital stay in patients undergoing VATS [12]. These findings were related to reduced pain, postoperative nausea and vomiting, emergence agitation, and opioid consumption following dexmedetomidine administration, making it a suitable choice for patients undergoing VATS.

These characteristics of dexmedetomidine are just what non-intubated VATS requires. Therefore, our study retrospectively determined the perioperative outcomes in 100 patients who underwent non-intubated VATS with thoracic epidural analgesia, intercostal nerve block, propofol, and either dexmedetomidine or alfentanil.

Doo et al. reported a prevalence of dexmedetomidine-induced hemodynamic instability of 14.7% in patients administered at a loading dose of 1 μg/kg, followed by 0.2–0.6 μg/kg/hr. for sedation. Female sex and obesity were found to be associated with a higher probability of developing dexmedetomidine-induced hemodynamic instability [13]. Hypotension occurred more frequently when dexmedetomidine was rapidly titrated [14]. Taittonen et al. suggested that premedication dose with 0.33–0.67 mg/kg IV given 15 min before surgery could minimize side effects of hypotension and bradycardia [15]. Therefore, in our study, dexmedetomidine infusion was administered at a very slow rate, with loading dose at 0.5 μg/kg for 30 min and then maintenance dose at 0.5 μg/kg/hr. until the surgery finished. Hence, the duration of anesthetic induction was longer in group D than in group O. Compared to group O, group D showed a significant decrease in heart rate, systolic blood pressure, diastolic blood pressure, and mean arterial pressure, from baseline values, and maintained a relatively stable hemodynamic status. In addition to the side effects of dexmedetomidine, the effects of vasodilation by propofol and thoracic epidural analgesia might not be excluded due to hypotension and bradycardia in both groups [16, 17]. Adequate pain control can prevent elevated blood pressure and accelerated heart rate [18]. Fluid challenge and ephedrine were administered for treatment.

Intraoperative one-lung arterial blood gas revealed lower pH and significant CO_2_ retention in group O, which indicates the preservation effects of CO_2_ elimination and the respiratory function of dexmedetomidine in group D. Although dexmedetomidine infusions did not result in clinically significant respiratory depression, sedation with dexmedetomidine reduced both hypoxic and hypercapnic regulation of breathing to a similar extent as sedation with propofol [19]. In addition, dexmedetomidine-induced sedation causes upper airway obstruction and episodes of apnea to the same degree as propofol-induced sedation [20]. Therefore, the respiratory condition and expiratory ETCO_2_ were closely monitored in our study. Pre-one lung arterial blood gas data obtained after prescribing midazolam and loading dose of dexmedetomidine revealed no significant difference compared to the alfentanil group premedicated with midazolam.

We routinely applied THRIVE for the entire duration of non-intubated VATS. One-lung ventilation during non-intubated VATS carries the risk of hypoxemia and hypercapnia, whereas THRIVE increases the oxygenation reserve status and efficiency of CO_2_ elimination [21]. Increased oxygenation and CO_2_ elimination reduce the respiratory dead space owing to the higher flow of oxygen [22–24]. In our study, pre-one-lung and one-lung oxygenation was similar between the two groups.

PONV, defined as occurring during the first 24 to 48 hours after surgery, is the most frequent side effect after general anesthesia and the least desirable outcome following surgery [25]. For enhanced recovery after surgery, Batchelor et al. in 2019 recommended a multimodal pharmacological approach for PONV prophylaxis for patients at moderate risk or high risk, and the administration of dexamethasone to prevent PONV [26]. The incidences of vomiting and nausea are approximately 30 and 50%, respectively, which may be as high as 80% in high-risk patients [27]. Patients with PONV may require unanticipated hospital admission and delayed recovery room discharge [28, 29]. In our study, eight patients suffered from PONV, which included one patient in group D and seven patients in group O. PONV high-risk patients should be predicted early using the Apfel score, and prokinetic premedication should be considered [30]. Serotonin receptor antagonists are effective in preventing and treating PONV with minor side effects. It binds to the serotonin receptor in both the vagal afferents of the gastrointestinal tract and the chemoreceptor trigger zone. Besides PONV, other common opioid-related side effects such as dyspnea, constipation, dizziness, and skin itching were also noted. In a meta-analysis by Ohishi et al., median occurrence rates of somnolence and dizziness in opioid-treated patients were 21% (range: 10–39%) and 22% (range: 10–37%), respectively [31]. In our study, dizziness was significantly more common in group O than in group D (group D vs. group O: 0 vs. 4, *p* < 0.05). Prochlorperazine was prescribed in ordinary wards postoperatively for these patients. The occurrence of these side effects might be the reason why patients in group O had a significantly prolonged postoperative hospital stay than those in group D.

According to the Enhanced Recovery After Surgery (ERAS®) protocol, regional anesthesia has a high level of evidence and is strongly recommended with the aim of reducing postoperative opioid use. A combination of acetaminophen and NSAIDs should be administered regularly to all patients unless contraindications exist [26]. Following non-intubated thoracoscopic lobectomy, Hung et al. in 2015 reported the administration of postoperative analgesia either by continuous epidural infusion of bupivacaine 0.1% and fentanyl (1.25 μg/mL) or by patient-controlled analgesia with intravenous morphine (1 mg/mL) for 2–3 days. Additional nonsteroidal analgesics were administered once patients resumed oral intake 2–4 hours after surgery. Complications of vomiting requiring medication were also recorded [16]. Dexmedetomidine has been proven to reduce perioperative opioid consumption and postoperative pain intensity [6, 32].

Our protocol was designed to control pain by intraoperative loading, postoperative single-dose fentanyl (50 μg) and bupivacaine 0.25% for maintenance through thoracic epidural analgesia, intraoperative intercostal nerve block as well as postoperative opioid and adjuvant analgesics, such as NSAIDs. Thoracic epidural placed before surgery has traditionally been the gold standard for achieving good analgesia acutely. Thereby, it reduces the likelihood of post-thoracotomy pain syndrome development and improves pulmonary mechanics and function by reducing splinting in thoracic surgery [33]. Another option is the intercostal nerve blockade [34], which has been considered a superior alternative to thoracic epidural if combined with a multimodal pain control regimen [35]. Morphine was administered in the postoperative room in our regimen when the numerical rating scale was rated higher than three points by patients. The consumption of morphine, diclofenac tablets, and Traceton® tablets was significantly decreased in group D, and the patients tolerated the treatment well without complications.

In 2015, Hung et al. reported that 13 patients converted to intubation and 1 patient converted to thoracotomy in non-intubated thoracoscopic lobectomy for lung cancer. Air leakage, subcutaneous emphysema, bleeding, and pulmonary and cardiovascular complications were also recorded [15]. In a retrospective study by Liu et al. in 2021, two patients were converted to multiportal incisions from uniportal thoracoscopic segmentectomy because of severe adhesion in the pleural cavity during the operation. In addition, one patient had prolonged air leakage [36]. In our study, there was no conversion to thoracotomy or multiportal incision. Surgical complications, including subcutaneous emphysema, air leakage, lung atelectasis, pneumothorax, and fever, were recorded, which were usually resolved or treated without sequelae. Patients were discharged earlier in group D than in group because of lesser opioid related discomforts. Oral NSAIDs tablets were prescribed for continuous OPD pain control.

This study has several limitations. First, this retrospective study evaluated anesthesia records of 100 patients between December 2016 and May 2022, which was conducted at a single-center, where statistical power might be limited by sample size. Second, patients in this study were sedated with either one of the two anesthesia regimens, selected by the anesthesiologist on duty. However, we applied a propensity bipartite matching score to approximate a random experiment that eliminates problems and selection bias associated with observational data analysis. Third, both techniques might not be equally distributed during the study period. Non-intubated VATS was a relatively new technique; thus, it would be an improvement in surgical and anesthesia techniques with time. Due to these limitations, prospective randomized controlled trials are required for further validation.

## Conclusion

In conclusion, the application of dexmedetomidine in non-intubated VATS in this retrospective study found a significant reduction in perioperative opioid-related complications, such as PONV, dyspnea, constipation, dizziness, and skin itching, and maintenance of spontaneous breathing with acceptable hemodynamic performance. The average anesthetic induction duration was longer owing to the loading time of dexmedetomidine. These clinical outcomes may enhance patient satisfaction and shorten the hospital stay. The confirmation of causal relationships would require further prospective randomized controlled trials.

## Data Availability

The datasets generated and/or analysed during the current study are not publicly available due to privacy or ethical restrictions, but are available from the corresponding author on reasonable request.

## Abbreviations

ASA: American Society of Anesthesiologists
BIS™: Bispectral Index™
BMI: Body mass index
CO_2_: Carbon dioxide
COPD: Chronic obstructive pulmonary disease
DLCO: Diffusing capacity for carbon monoxide
EKG: Electrocardiography
ETCO_2_: End-tidal carbon dioxide
FEV1: Forced expiratory volume-one second
FiO_2_: Fraction of inspired oxygen
FVC: Forced vital capacity
IV: Intravenous
NIBP: Non-invasive blood pressure
NSAIDs: Nonsteroidal anti-inflammatory drugs
OPD: Outpatient clinic
PaCO_2_: Partial pressure of carbon dioxide
PaO_2_: Partial pressure of oxygen
PONV: Postoperative nausea and vomiting
PSM: Propensity score matching
SpO_2_: Saturation of peripheral oxygen
TCI: Target-controlled infusion
THOPAZ: Thopaz Digital Chest Drainage System
THRIVE: Transnasal humidified rapid-insufflation ventilatory exchange
VATS: Video-assisted thoracic surgery
